# Deguelin Attenuates Reperfusion Injury and Improves Outcome after Orthotopic Lung Transplantation in the Rat

**DOI:** 10.1371/journal.pone.0039265

**Published:** 2012-06-20

**Authors:** Patrick Paulus, Pia Ockelmann, Sabine Tacke, Nora Karnowski, Peter Ellinghaus, Bertram Scheller, Johannes Holfeld, Anja Urbschat, Kai Zacharowski

**Affiliations:** 1 Clinic of Anesthesiology, Intensive Care Medicine and Pain Therapy, Goethe-University Hospital Frankfurt, Frankfurt am Main, Germany; 2 Department of Veterinary Clinical Sciences, Clinic for Small Animals Surgery, Justus-Liebig University, Giessen, Germany; 3 Clinical Pharmacology, Global Biomarker, Bayer Pharma AG, Wuppertal, Germany; 4 Clinic of Cardiac Surgery, Innsbruck Medical University, Innsbruck, Austria; 5 Clinic of Urology, Goethe-University Hospital Frankfurt am Main, Germany; Medical University Innsbruck, Austria

## Abstract

The main goal of adequate organ preservation is to avoid further cellular metabolism during the phase of ischemia. However, modern preservation solutions do rarely achieve this target. In donor organs hypoxia and ischemia induce a broad spectrum of pathologic molecular mechanisms favoring primary graft dysfunction (PGD) after transplantation. Increased hypoxia-induced transcriptional activity leads to increased vascular permeability which in turn is the soil of a reperfusion edema and the enhancement of a pro-inflammatory response in the graft after reperfusion. We hypothesize that inhibition of the respiration chain in mitochondria and thus inhibition of the hypoxia induced mechanisms might reduce reperfusion edema and consecutively improve survival *in vivo*. In this study we demonstrate that the rotenoid Deguelin reduces the expression of hypoxia induced target genes, and especially VEGF-A, dose-dependently in hypoxic human lung derived cells. Furthermore, Deguelin significantly suppresses the mRNA expression of the HIF target genes VEGF-A, the pro-inflammatory CXCR4 and ICAM-1 in ischemic lungs vs. control lungs. After lung transplantation, the VEGF-A induced reperfusion-edema is significantly lower in Deguelin-treated animals than in controls. Deguelin-treated rats exhibit a significantly increased survival-rate after transplantation. Additionally, a downregulation of the pro-inflammatory molecules ICAM-1 and CXCR4 and an increase in the recruitment of immunomodulatory monocytes (CD163+ and CD68+) to the transplanted organ involving the IL4 pathway was observed. Therefore, we conclude that ischemic periods preceding reperfusion are mainly responsible for the increased vascular permeability via upregulation of VEGF. Together with this, the resulting endothelial dysfunction also enhances inflammation and consequently lung dysfunction. Deguelin significantly decreases a VEGF-A induced reperfusion edema, induces the recruitment of immunomodulatory monocytes and thus improves organ function and survival after lung transplantation by interfering with hypoxia induced signaling.

## Introduction

Lung transplantation is, despite the highest mortality among all solid organ transplants, the only therapeutic option in patients with end-stage pulmonary disease [1]. The fragility and the poor tolerance against ischemia of this organ is responsible for the fact that only 20% of the candidate lungs are currently being transplanted [2]. Today, primary graft dysfunction (PGD) represents the most common acute complication during the first 30 days after lung transplantation [1], [3]. PGD mainly develops on the soil of an ischemia-reperfusion (IR) reaction that is characterized by an overshooting inflammatory response, culminating in tissue edema and consecutive graft failure [4], [5].

Despite downregulation of the cellular metabolism by modern preservation methods (i.e. preservation solutions, hypothermia etc.), hypoxia- induced gene transcription is not completely blunted. However, the complete blockade of the cellular metabolism for the time of ischemia might be of benefit. First, due to the loss of oxygen consumption, no metabolites are produced, that might have an adverse effect on cellular viability. Second, upon inhibition of cellular respiration, the intracellular oxygen concentration remains normal under ischemia, and thus oxygen is available for hydroxylation of the transcription factor *Hypoxia Inducible Factor* (HIF)-1, thus resulting in its decreased transcriptional activity [6], [7], [8].

HIF-1 occupies a central and critical role, involving a large number of genes [9]. Among these genes, especially *Vascular Endothelial Growth Factor* (VEGF) and genes that regulate cell adhesion to the endothelium such as *Inter-Cellular Adhesion Molecule* (ICAM)-1 are activated within minutes by HIF-1 [10], [11], [12], [13], [14], [15]. VEGF is known to quickly increase vascular permeability about 50.000 times stronger than histamine, by enhancing the break-down of the endothelial barrier function and inducing the formation of interendothelial gaps [16]. This in turn leads to an increased tissue edema ending up in organ failure [14], [17], [18], [19], [20], [21], [22], [23]. Together with ICAM-1, VEGF is highly chemotactic for inflammatory cells like monocytes [24], [25].

Clinically relevant effects of hypoxia induced pathomechanisms are observed in high-altitude mountaineers, where low oxygen tension is highly correlated with lung and brain edema, as well as systemic inflammatory response syndrome [26], [27], [28], [29]. Hypoxia thus triggers inflammation, and on the other hand, inflammation itself causes local hypoxia, combined with activation of the coagulation system [19], [30], leading to a vicious circle as seen in acute lung injury [31], [32].

After restoration of the normal blood flow (reperfusion), the resulting increased concentration of pulmonary VEGF leads to an increased vascular permeability resulting in early post transplantation dysfunction [33], [34], [35], [36], [37].

However, the main functions of the hypoxia induced genes are meant “self-protective=" by preventing organ damage (i.e. increase of hemoglobin, increase of micro-vessel density through neo-angiogenesis, increase of glycolysis to ensure cellular metabolism). Unfortunately, at least in endothelium-rich tissues, these effects seem to have a negative impact on the outcome after ischemic events i.e. transplantation. It has been shown that these negative effects are mainly triggered by HIF-1 and thus correlate with primary graft failure [14], [15], [22], [38]. A dual blockade of the respiration chain and the hypoxia induced gene expression might therefore be of benefit in lung transplantation.

The rotenoid Deguelin is able to provide this dual inhibition. Deguelin acts as a mitochondrial inhibitor of the respiration chain via inhibition of the mitochondrial NADH dehydrogenase/complex I [39]. The resulting reduced oxygen consumption leads to increased HIF-1 hydroxylation and thus inhibition of its transcriptional activity [7]. Therefore, a blockade of the deleterious effects of HIF-1 during lung transplantation might be beneficial to prevent PGD and improve short-term survival by reducing tissue edema.

## Results

### Deguelin Gavage is Well Tolerated

**Figure 1:**
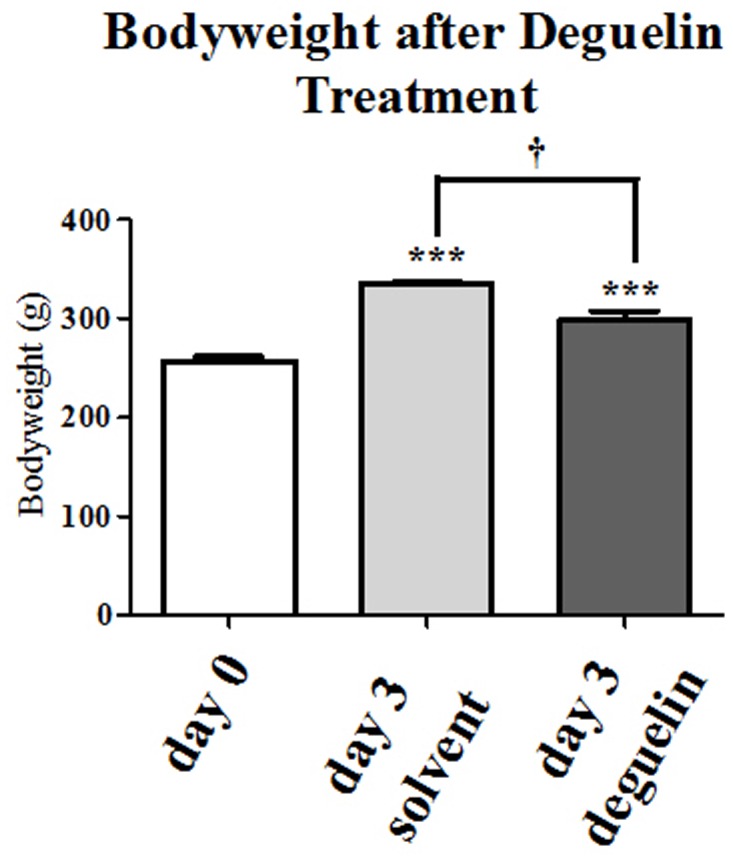
Deguelin gavage is well tolerated. Bodyweight is determined before and after Deguelin treatment. The gain in bodyweight is a sign for good tolerance of the substance. The graph represents the increases in bodyweight vs. weight at begin of the experiment. Columns and error bars represent means ± SEM. †, P<0.02; ***, P≤0.0009; one-way ANOVA and unpaired t test.

All animals received two daily gavages of Deguelin intra-gastrically at 4 mg/kg bodyweight for 3 days. All animals treated with Deguelin or solvent significantly gained weight during the trial (297.8±9.805 g and 335.6±2.064 g vs. 257.1±4.389 g, P = 0.0009, P<0.0001 resp.). However, animals receiving only solvent, were significantly heavier compared to the Deguelin treated animals (335.6±2.064 vs. 297.8±9.805, †P = 0.0196). No deleterious side effects were observed in all groups (Fig. 1).

### Deguelin Effectively Blocks HIF-1 during Hypoxia *in vitro*

**Figure 2:**
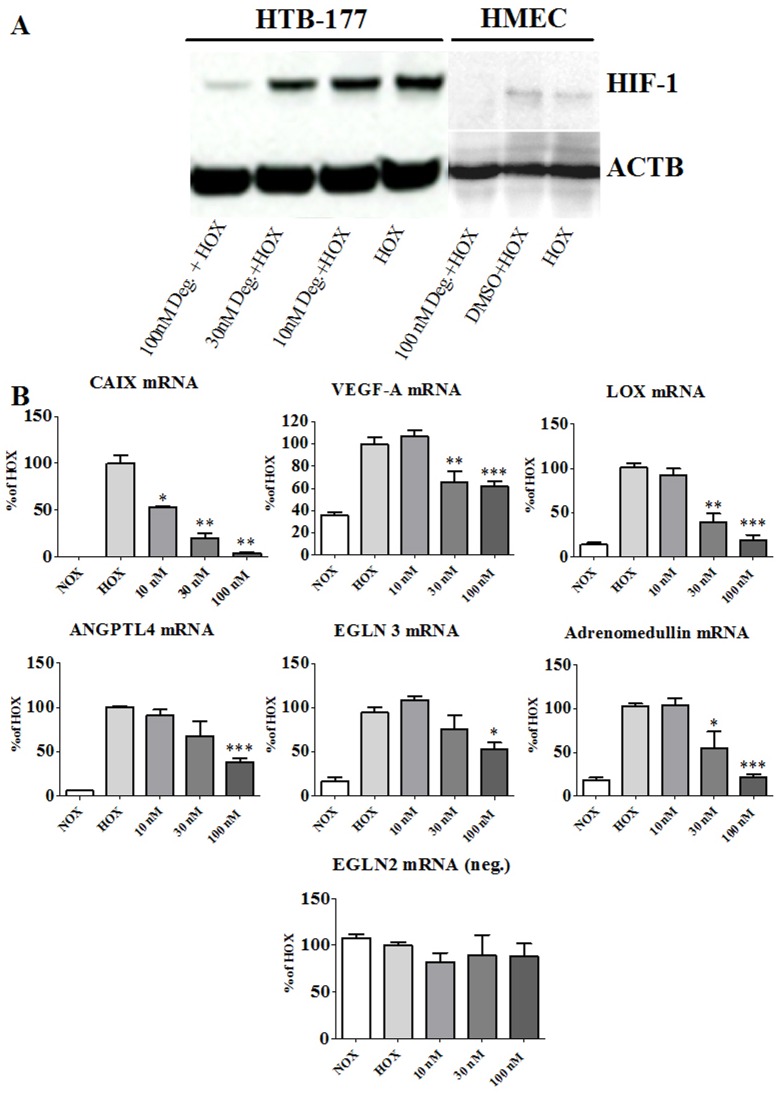
Deguelin effectively inhibits HIF-1 and its reporter genes during hypoxia *in vitro*. Human lung epithelial derived and endothelial cells are incubated for 6 hours under hypoxia. Different concentrations of Deguelin are tested to determine a concentration dependent inhibition of HIF-1 induced genes. (A) Western blot analysis of lung derived epithelial cells (NCI-H460 and HTB-177) and human microvascular endothelial cells (HMEC) revealed the concentration dependent suppression of HIF-1 by Deguelin. Beta-Actin (ACTB) serves as internal negative control. (B) Graphs representing mRNA expression profiles of HIF-1 regulated genes: concentration dependent inhibition HIF-1 induced target genes. EGLN2 expression serves as internal negative control. NOX = normoxia, HOX = hypoxia. Measurements were performed in triplicate. Columns and error bars represent means ± SEM. * indicates significance level vs. HOX; *, P<0.05; **, P≤0.01; ***, P<0.0005; one-way ANOVA and unpaired t test.

Deguelin effectively suppressed HIF-1 protein after 6 hours hypoxia *in vitro* at a concentration of 100 nM in human microvascular endothelial cells (HMECs) as well as in human lung epithelial derived cells (NCI-H460 and HTB-177). DMSO (solvent) with hypoxia and hypoxia (HOX) alone stabilized HIF-1(Fig. 2A). Deguelin effectively inhibits the hypoxia-induced expression of HIF target genes in a concentration dependent manner. Carbonic anhydrase IX (CAIX), vascular endothelial growth factor (VEGF)-A, lysyl oxidase (LOX), angiopoietin-related protein 4 (ANGPTL4), egl nine homolog 3 (EGLN3) and adrenomedullin (ADM) mRNA expression is significantly downregulated in hypoxic cells (HOX) upon treatment (Fig. 2B). EGLN2, a HIF prolyl hydroxylase that is not a HIF target gene serves as negative control, to show that Deguelin is not a general transcription inhibitor.

### Deguelin Effectively Blocks HIF-1 during Hypoxia *in vivo*

**Figure 3:**
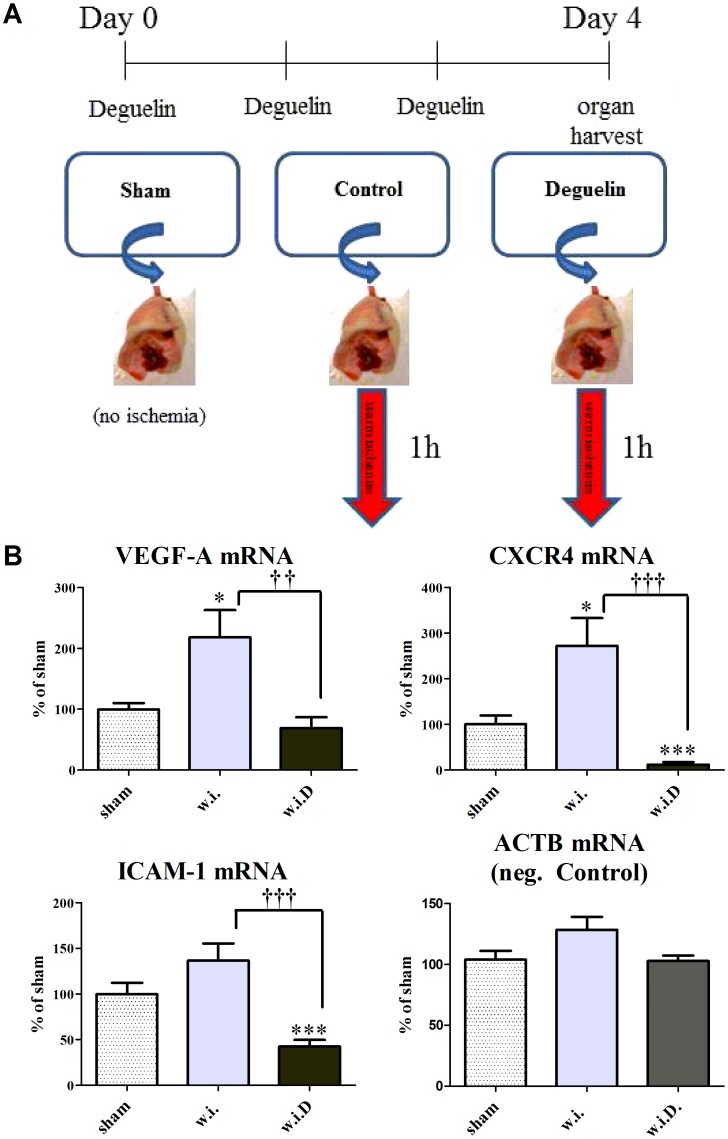
Deguelin effectively inhibits pro-edema and pro-inflammatory genes during hypoxia *in vivo*. Lungs from animals pretreated with or without Deguelin are explanted. After 1 hour incubation at 37°C, simulating warm ischemia, the ischemic lungs are processed for further analysis. (A) Scheme representing the ischemia experiment. (B) Gene expression of VEGF-A, CXCR4 and ICAM-1 in ischemic lungs treated with or without Deguelin. Beta actin served as negative control. Groups are compared to native Lungs (sham). Sham  =  native lungs without ischemia, w.i.  =  ischemic lungs without treatment, w.i.D  =  ischemic lungs with Deguelin treatment. Measurements were performed in triplicate. Columns and error bars represent means ± SEM. * indicates significance level vs. sham, † indicates significance level vs. w.i.; *, P<0.05; ***, P<0.0005; ††, P<0.005; †††, P<0.0005; one-way ANOVA and unpaired t test.

To test whether Deguelin has a sufficient *in vivo* activity, treatment was initiated 3 days prior to sacrification at 4 mg/kg BW twice/day. On day 4 the animals were killed and the lungs were excised and stored at 37°C for 1 h, simulating warm ischemia (w.i.). Sham lungs were immediately snap-frozen without ischemia (Fig. 3A). The readout was performed on these samples by measuring HIF-1 regulated genes using RT-PCR. Basal mRNA expression under normoxia (sham) was detectable for VEGF-A (100.0±12.28), CXCR4 (100.0±19.97) and ICAM-1 (99.9±12.28). Under warm ischemia, VEGF-A (217.8±44.98; P = 0.0338) and CXCR4 (272.0±60.82; P = 0.0291) were significantly upregulated vs. sham, whereas the changes in ICAM-1 (136.5±18.93; n.s.) gene expression were not significant. Deguelin treatment (w.i.D.) significantly blunted VEGF-A (68.8±17.90; P = 0.0042), CXCR4 (11.9±5.53; P = 0.0003) and ICAM-1 (42.4±7.36; P<0.0001) mRNA levels compared to warm ischemia. The gene expression of the corresponding negative control β-Actin (ACTB) remained unchanged (Fig. 3B). These results indicate that Deguelin potently interferes with the hypoxia induced gene expression (Fig. 2B). Especially VEGF-A, which regulates vascular permeability, and ICAM-1 as well as CXCR4, two genes regulating leukocyte invasion and consequently edema enhancement, are suppressed by systemic Deguelin treatment.

### Deguelin Prevents from Structural Changes and Cellular Edema in Ischemic Lungs

**Figure 4:**
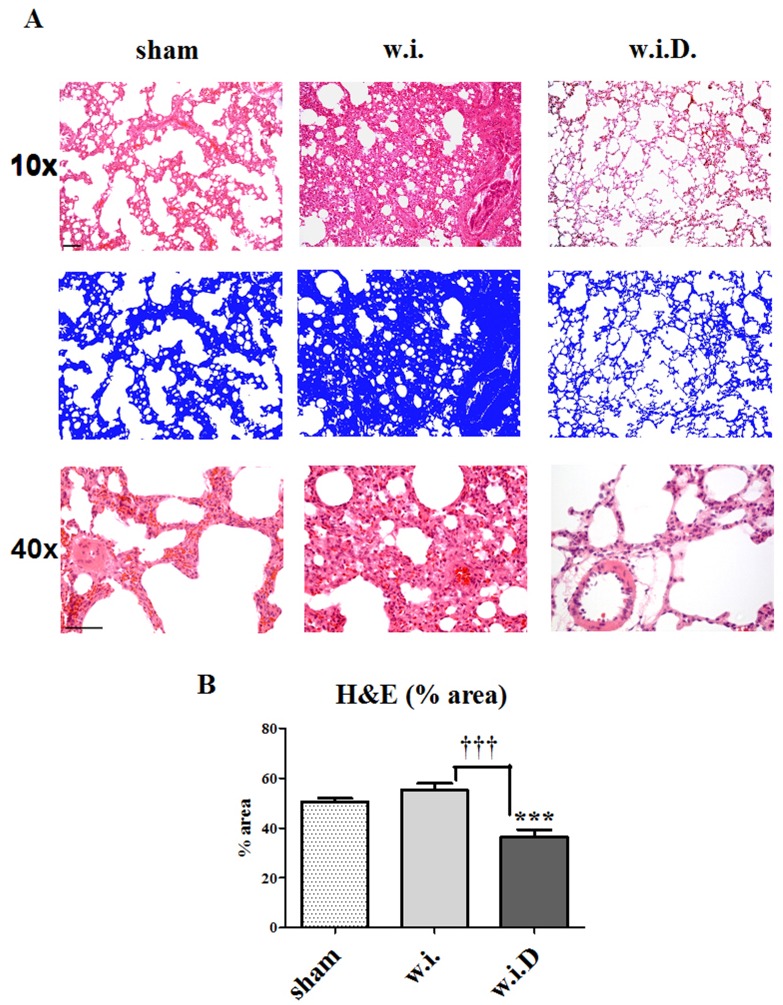
Deguelin prevents from ischemia induced edema and loss of lung microstructure. From the explantation experiment, micrographs are analyzed to detect structural edema as sign for organ damage. (A) Representative H&E micrographs (purple) from native lungs (sham), ischemic lungs without treatment (w.i.) and ischemic lungs with deguelin treatment (w.i.D). The blue graphs represent planimetric evaluation of the H&E stains to evaluate the area/field occupied by tissue as measurement for edema. Magnifications used: 10× and 40×. Bar in the 10× magnified micrographs represent 100 µm and in the 40× magnified micrographs represent 50 µm. From each H&E stain representing always one animal, 3 different areas were photographed and evaluated. (B) Graph representing the evaluation of the planimetric measurements. Columns and error bars represent means ± SEM. * indicates significance level vs. sham, † indicates significance level vs. w.i.; ***, P<0.0005; †††, P<0.0001; one-way ANOVA and unpaired t test.

From the organs stored at 37°C for 1 h, samples were collected and histology was performed. All sections were stained with H&E and pictures with 10× and 40× magnification were taken. To quantify cellular edema and structural pulmonary changes, the area occupied by tissue (positive H&E staining) was determined planimetrically. For this purpose, 3 randomly taken pictures from each slice were analyzed (Fig. 4A, blue). The increase in cellular edema and structural changes directly correlate with the increase in area occupied by lung tissue per section. Lungs from animals that received Deguelin (w.i.D.) prior to ischemia (36.5±2.88% tissue/area), displayed significantly reduced cellular edema and structural changes than those from sham (50.5±1.34% tissue/area) or with warm ischemia (w.i.) (55.2±2.73% tissue/area) groups (Fig. 4B). In animals receiving Deguelin (w.i.D.), the microstructure was preserved. These results indicate that hypoxia/ischemia mediate the development of acute tissue edema.

### Deguelin Treatment Reduces Reperfusion Edema by Suppression of VEGF-A Protein in Lung Grafts and Improves Short-term Survival after Orthotopic Lung Transplantation

**Figure 5:**
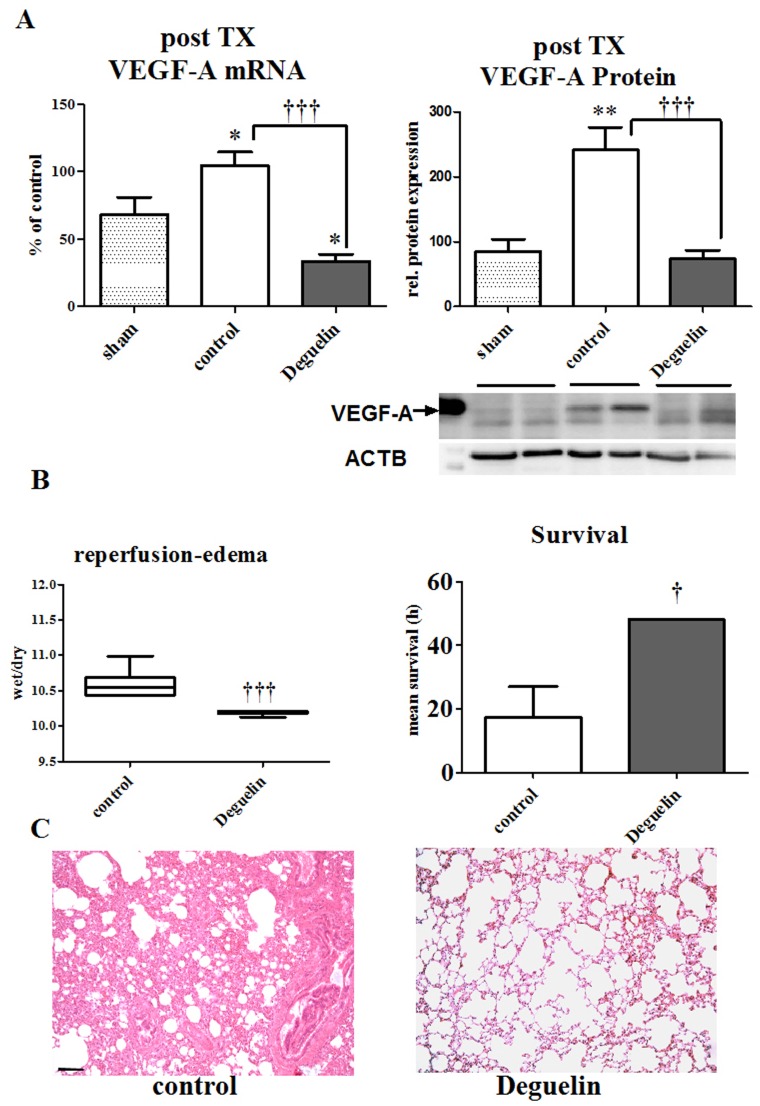
VEGF-A activity correlates with edema formation. The transplantation experiments compromise two groups, one control (perfadex only) and one Deguelin treated group. Animals receiving Deguelin (donor and recipient) are pretreated 3 days prior transplantation and treatment is kept upright in the recipients until the end of the experiment after 48 hours after transplantation. (A) Graphs and representative western blot images representing tissue VEGF-A mRNA and protein levels after transplantation and reperfusion. Animals that received Deguelin are compared vs. controls. (B) Right graph representing the wet-to-dry ratio evaluating the extent of tissue edema. Left graph representing the mean survival expressed in hours of both groups (controls vs. animals that received Deguelin). (C) Corresponding micrographs (10× magnification, H&E) show microstructural changes that occur after reperfusion. Calibration bar represents 100 µm. Columns and error bars represent means ± SEM. * indicates significance level vs. control. ***, P<0.0005; one-way ANOVA and unpaired t test.

To test the hypothesis whether Deguelin might reduce tissue edema *in vivo* via modulation of the VEGF-A pathway, we performed transplantation experiments. For this purpose we transplanted the left lung from either pre-treated donors to pre-treated recipients (Deguelin) or the left lung from non-treated donors to non-treated recipients (control). Analysis of the transplanted organs revealed that lungs from animals, which were treated with Deguelin, had significantly lower levels of VEGF-A mRNA and protein when compared to untreated subjects (Fig. 5A).

A small part of the transplanted left lung was used to determine the wet-to-dry ratio, directly reflecting the extent of reperfusion edema in the lung tissue. Animals, which received Deguelin, had significantly less tissue water and thus less edema compared to lungs that did not receive any treatment (Fig. 5B left graph). Together with these findings, animals that had lower post-transplantation-edema also had a significant better survival rate. 100% of the Deguelin treated animals (Deguelin) survived until the end of the observation period of 48 h, whereas controls only showed a mean survival of 17.42±9.672 hours (P = 0.0101; Fig. 5B right graph). The better survival therefore directly correlates with the blunted VEGF-A expression, showing the direct correlation of VEGF-A and the development of tissue edema and organ dysfunction.

In corresponding micrographs, lungs that have a high degree of edema show similar structural changes compared to lungs from the preliminary experiment (Fig. 5C) that underwent warm ischemia, without administration of Deguelin. These data suggest, that hypoxia directly induces reperfusion edema via the upregulation of VEGF-A expression.

### Deguelin Treatment Diminishes the Pro-inflammatory Tissue Response and Stimulates Immunomodulation via Recruitment of Anti-inflammatory M2 Macrophages

**Figure 6:**
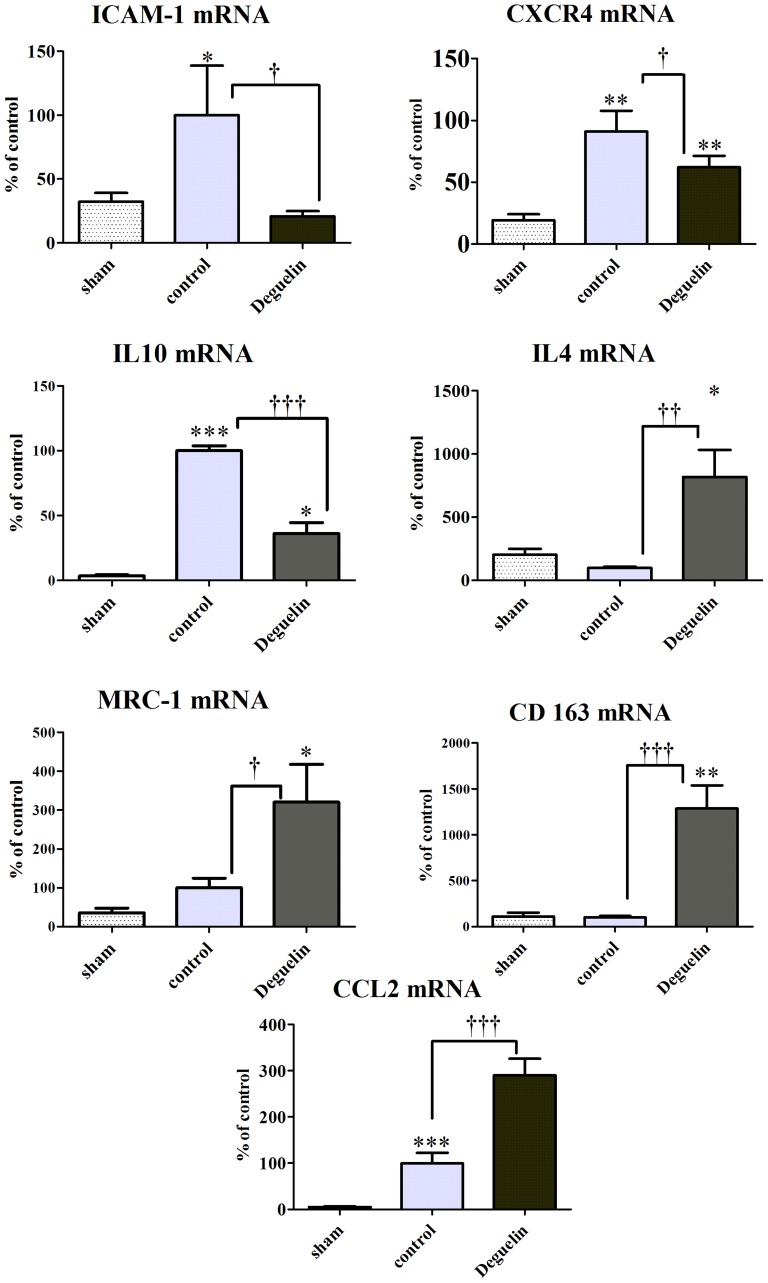
Hypoxia and reperfusion induce inflammatory gene expression. 48 hours after transplantation, the graft is analyzed for hypoxia induced pro-inflammatory pathways via RT-PCR. Graphs representing the mRNA expression of hypoxia induced inflammatory mechanisms (ICAM-1 and CXCR4) and mRNA expression of markers of anti-inflammatory monocytic cell recruitment in lungs at the end of the reperfusion phase. Columns and error bars represent means ± SEM. † indicates significance level vs. controls; * indicates significance levels vs. sham; one-way ANOVA and unpaired t test.

Deguelin protects from acute inflammation by downregulating pro-inflammatory peptides such as ICAM-1 and CXCR4. In Deguelin treated animals, tissue expression of the pro-inflammatory genes, directly regulated by hypoxia, is blunted as a result of the treatment. ICAM-1 (26.5±5.3 vs. 127.8±49.6; P = 0.0188) and CXCR4 (58.9±8.7 vs. 107.7±20.6; P = 0.0274) gene expression is significantly downregulated in Deguelin treated animals vs. controls. In contrast, tissue mRNA expression of markers related to the recruitment of anti-inflammatory macrophage populations such as IL4 (153.1±14.5 vs. 100.0±17.2; P = 0.0357), mannose receptor C type 1 (MRC-1) (320.5±96.6 vs. 100.0±24.9; P = 0.044), CCL2 (290.0±35.7 vs. 99.9±22.4; P = 0.0001) and CD 163 (1262.0±266.6 vs. 100.0±18.5; P = 0.0008) is significantly upregulated in Deguelin treated animals vs. controls. These findings indicate that in this *in vivo* model, the recruitment of M2 macrophage populations is CCL2, IL4 and MRC-1 dependent (Fig. 6). Concerning IL10, our data show a downregulation in Deguelin treated animals vs. controls (36.18±8.323 vs. 100.0±3.801; P<0.0001).

**Figure 7:**
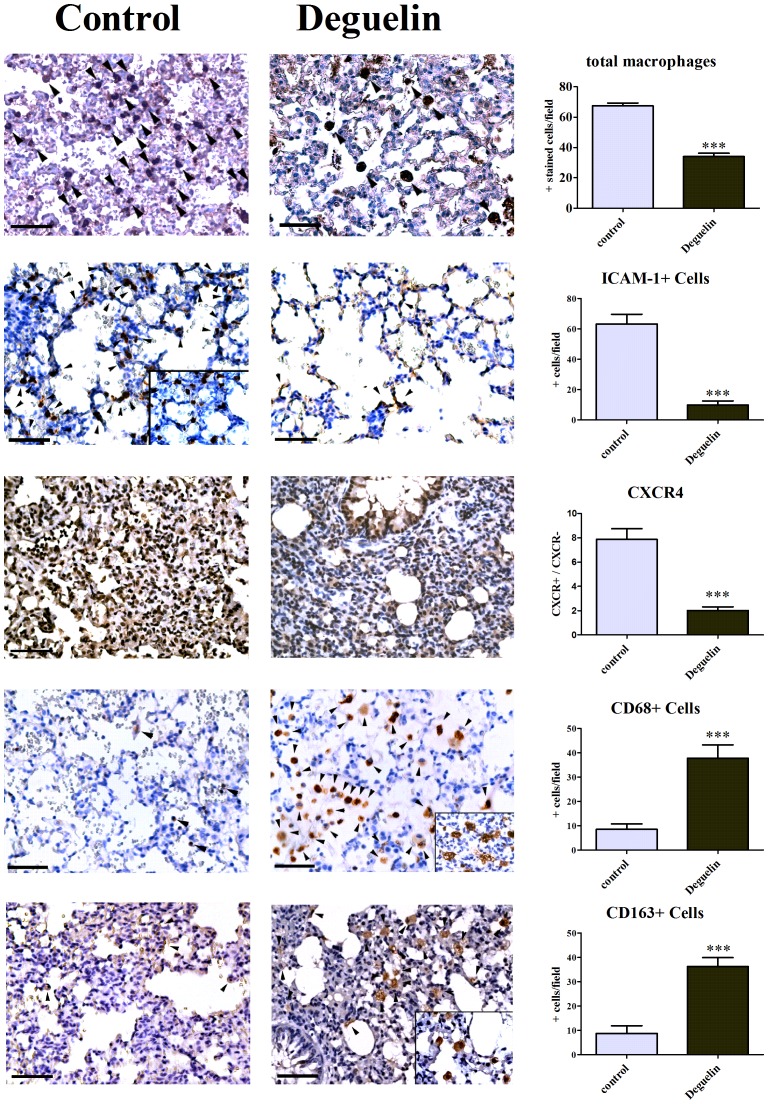
Anti-inflammatory macrophages are recruited to Deguelin treated lungs. Figure representing micrographs of cellular invasion into transplanted lungs at the end of the reperfusion phase. The left micrographs represent control animals and the right micrographs represent animals that received Deguelin treatment. From top to bottom, micrographs represent DAB immunostainings from RM-4 (pan-macrophage), ICAM-1, CXCR4, CD68 and CD163. Magnification was set at 40× and 100× for pictures in picture. CXCR4 micrographs are represented in 20× magnification. Calibration bar represents 50 µm (40×). The graphs represent the statistical evaluation of each cell type. The upper graph represents a total macrophage count (RM-4+ cells), followed by ICAM-1+ cell count and CXCR4staining. The last two graphs represent CD68+ cell count, and finally a CD 163+ cell count. Arrowheads mark positively stained cells. Evaluation for CXCR4 is performed by calculating the ratio between positively and negatively stained amount of cells to avoid bias from alveoli that contain no cells. Arrowheads mark positively stained cells. Columns and error bars represent means ± SEM. * indicates significance level vs. control. ***, P<0.0001; one-way ANOVA and unpaired t test.

Besides, Deguelin also has immunomodulatory effects on the donor lung by favoring the recruitment of anti-inflammatory macrophages (CD 163+) involving the CCL2/MRC-1/IL4 pathway. In corresponding micrographs, the amount of CD 163+ cells is significantly higher in animals that have been treated with Deguelin vs. controls (36.2±3.7 vs. 8.7±3.1; P<0.0001). This directly correlates with the better outcome of these animals. A CD 68 staining reveals that mature macrophages are mainly participating in this anti-inflammatory and HIF-1 inhibitory effect. In Deguelin treated animals the amount of CD 68+ cells is significantly higher than in non-treated animals (37.8±5.9 vs. 8.5±2.2; P<0.0001) and thus correlates directly with the amount of CD163+ cells. However, in a pan-macrophage/dendritic cell staining (RM-4), the total macrophage count is significantly higher in untreated than in Deguelin treated animals (67.4±1.9 vs. 34.0±2.3; P<0.0001). Furthermore, the amount of RM-4 positively stained macrophages in the Deguelin group corresponds to the amount of CD-163+ cells from the same group (Fig. 7).

Interestingly, it seems that the CD 68+ and CD 163+ cells are attracted via CCL2/MRC-1/IL4 rather than IL10 or ICAM-1 signals. ICAM-1 immunostaining shows that its expression is blunted in Deguelin treated animals (9.8±2.7 vs. 63.2±6.3; P<0.0001) and thus correlates with tissue mRNA ICAM-1 expression (Fig. 6, 7).

### IL6 Protein Downregulation and Interferon (IFN) γ Protein Upregulation is Co-associated with Adverse Outcome

**Figure 8:**
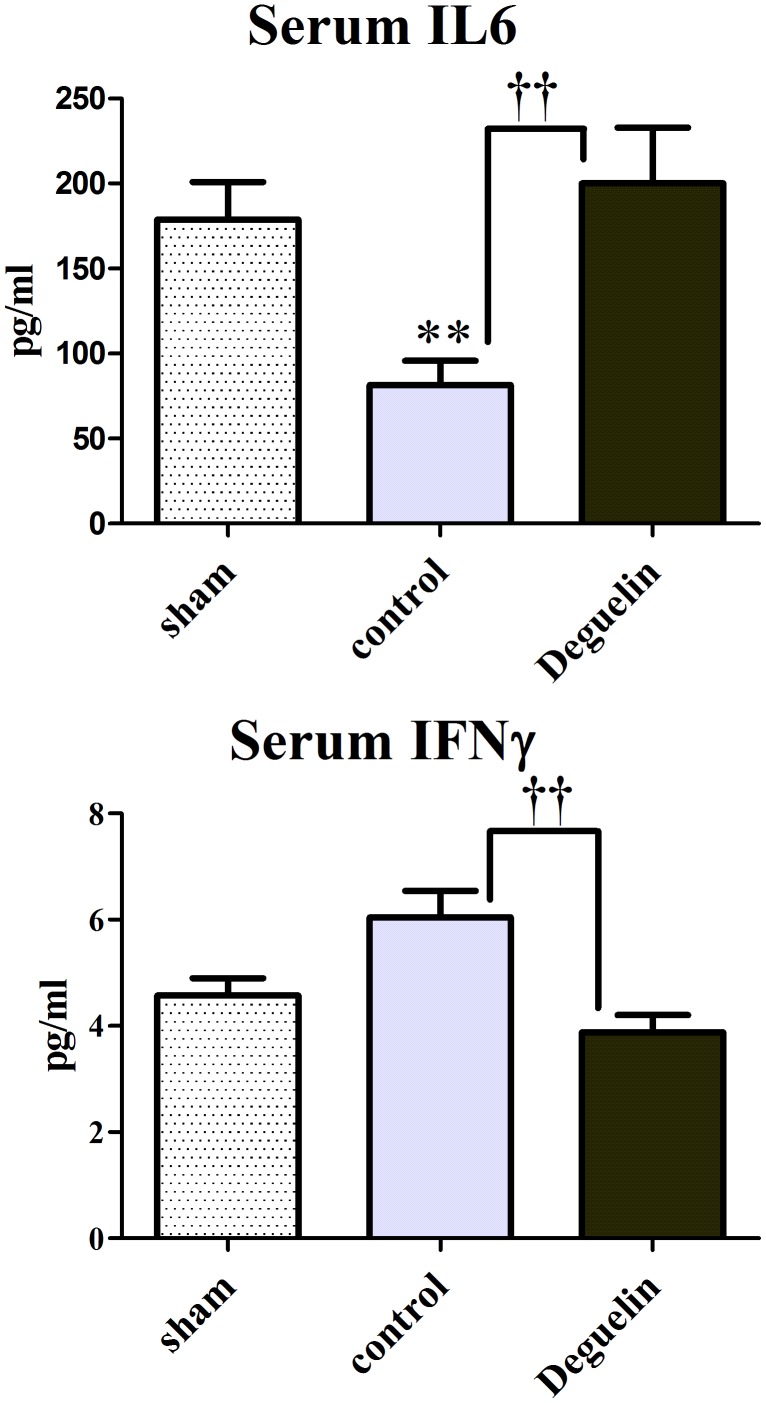
Low Serum IL6 and high Interferon (IFN) γ levels are associated with adverse outcome. Fig. 8 shows ELISA analyses of serum samples from sham, Deguelin and Perfadex (controls) treated animals from the transplantation experiment. IL6 is significantly blunted in controls vs. sham or Deguelin treated animals. The pro-inflammatory IFN γ is upregulated in controls vs. Deguelin or sham animals. Columns and error bars represent means ± SEM. † indicates significance level vs. controls; * indicates significance levels vs. sham; one-way ANOVA and unpaired t test.

IL6 and IFNγ are known to play an important role in inflammatory reactions especially in accordance to rejection reactions. As the recruitment of M2 macrophages also requires to some extent a pro-inflammatory reaction, we aimed to test whether IL6 or IFNγ might be regulated in our experimental setting. Therefore we performed ELISA on serum samples derived from sham, Deguelin and Perfadex-treated (control) rats. Interestingly, animals that have been treated with Perfadex only, have significantly lower levels of serum IL6 as seen in Deguelin or sham animals (81.5±14.3 pg/ml vs. 200.0±32.8 pg/ml resp.178.9±22.0 pg/ml; P<0.004). Consistent with this, the serum protein expression of IFNγ is significantly upregulated in controls vs. Deguelin treated animals (6.1±0.5 pg/ml vs. 3.9±0.3 pg/ml; P = 0.0025). These data show that both IL6 and IFNγ might play an important role in orchestrating these inflammatory reactions and showing that a lack of IL6 and an overexpression of IFNγ are co-associated with the M1 or pro-inflammatory macrophage phenotype (Fig. 8).

## Discussion

Today, the number of patients on the waiting lists for organ transplantation is steadily increasing; in contrast, the number of available organs is not. This leads to the fact that currently organs are considered for transplantation even when potentially subjected to longer ischemia periods or poor hemodynamics of the donor. Some years ago, they probably would have been rejected [40]. Given these circumstances, pharmacologic protection of the available organs is of increasing interest. Ischemia and reperfusion (IR) injury to organs represents one of the most common clinical problems in transplantation medicine. IR may lead to primary or secondary organ failure after restoration of the blood flow [41], [42], [43]. The theory that hypoxia triggers inflammation is not new. There is sufficient evidence that hypoxia leads to activation of several deleterious pathways like the occurrence of edema due to enhanced vascular leakage and accumulation of inflammatory cells in organs, increasing the risk of early and late graft rejection after transplantation [27]. Hypoxia or ischemia are known triggers of a vicious circle, enhancing inflammation and organ damage. Simplified, the paradigm - tissue hypoxia leads to inflammation, tissue inflammation leads to hypoxia- can be applied [44].

**Figure 9:**
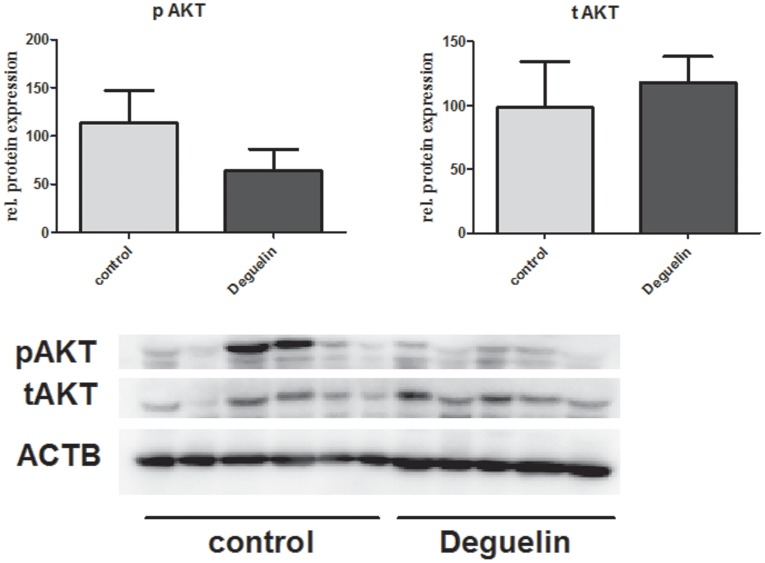
Specificity of Deguelin. Western blot analysis of total AKT (tAKT), phospho AKT (pAKT) and ACTB of transplanted lungs derived from animals that were either treated with Deguelin or without (control). However a trend to lower levels of pAKT (n.s.; P = 0.2403) may be seen, no significant differences are observed between the two groups, underlining HIF-1 downregulating potency of Deguelin.

During hypoxia or ischemia, HIF-1 is mainly responsible for the initiation of gene transcription in many lung diseases like gut-induced lung injury following IR, hypoxic pulmonary hypertension, COPD and fibrous airway remodeling [45], [46], [47], [48]. Hypoxia stabilizes HIF-1 which in turn directly and quickly upregulates target gene’s expression within minutes [49], [50], [51], [52]. Usually, the maximum transcription rate culminates at 2 hours of hypoxia. In the present study, however, we could demonstrate that HIF-1 induces transcription in ischemic lung tissue already much earlier (within 1 hour), however with lower efficacy. The reason for this quick response upon hypoxia might be explained by the fact that hypoxic organs and cells have to react immediately to prevent damage and ensure survival of the organism. Therefore we suppose that HIF-1 has two major actions, a deleterious fast and pro-edematous one, and a longer lasting one, namely angiogenesis and endothelial proliferation [17]. In transplantation medicine, VEGF has been shown to exert pro-inflammatory and pro-apoptotic actions during the early phase of reperfusion [53]. Our own data concerning patients who underwent lung or heart transplantation could correlate increased HIF-1 binding activity and high VEGF-A levels with more pronounced edema and rejection rates [22], [23], [37]. Therefore these findings led to the hypothesis that the inhibition of VEGF-A pathways, as well as other hypoxia triggered pro-inflammatory genes such as ICAM-1 and CXCR4 could reduce early complications such as edema-caused PGD after lung transplantation. Several recent publications also describe protective effects of the latter genes. Namely the work by Jiang et al. demonstrated that a therapeutic enhancement of the vascular integrity via HIF-1 activation during acute rejection promotes tracheal graft health and prevents from chronic rejection [54]. This work stands in direct contrast to the work of Krebs et al. who demonstrated that VEGF is responsible for the occurrence of bronchiolitis obliterans [55]. These data indicate that the sum of the hypoxia induced effects might be protective. One possible explanation might be that the work by Jiang et al. does not include short-term survival results reflecting early complications. As the trachea is not participating in the gas exchange, animals might show early inflammation, but they will not die of it, as both lungs are still functional and gas exchange remains unchanged. To test our hypothesis that the summative effects of hypoxia negatively influence the graft fate during the early reperfusion phase by increasing tissue edema and inflammation, we used the model of orthotopic lung transplantation in the rat. This model has the advantage that a read out of early complications by providing short term survival data is possible. In the present work, we could demonstrate that the summative effect of hypoxia induced gene expression is deleterious. We could show for the first time that solely 1 hour of hypoxia leads to an increase of pro-edematous VEGF-A mRNA expression and in turn leading to an edematous tissue alteration as shown in figure 4. Transplanted and reperfused lungs without Deguelin treatment had a high water content as depicted in the wet-to-dry ratio (Fig. 5B). Together with this, the survival was significantly increased in the Deguelin group, underlining the beneficial effect of HIF-1 Inhibition *in vivo*. The mechanisms by which Deguelin exerts the anti-inflammatory properties might rather directly result from inhibition of the HIF-1 pathway than from interference with the Pi3K/Akt pathway, which is not addressed by the concentration of deguelin used in this model (Fig. 9). Our data show for the first time that treatment with Deguelin significantly reduces hypoxia induced deleterious effects in lung grafts and thereby significantly lowers reperfusion edema and improves survival. These results indicate that systemic perioperative inhibition of the respiratory chain in mitochondria in donor organs might be a beneficial therapeutic option. It was shown recently that ICAM-1 and CXCR4 are involved in lung injury via recruitment of inflammatory cells [56], [57]. We could now demonstrate that Deguelin application *in vitro* and *in vivo* leads to a consecutive downregulation of ICAM-1 and CXCR4. This attenuation consequently leads to less inflammation in the grafts. When looking at the micrographs, we found however that Deguelin treatment leads to occupation of the alveolar spaces by monocytic cells. This however appears not logical, as the lung inflammation markers ICAM-1 and CXCR4 were downregulated. Immunohistochemical staining revealed that these monocytic cells highly expressed the surface markers CD 163, meaning that these monocytes are anti-inflammatory macrophages. The marker CD 68, which represents mature macrophages, is poorly expressed in healthy lungs, however in the transplanted lung CD 68 is upregulated indicating that the anti-inflammatory macrophages are fully differentiated. Whether these macrophages are recruited as precursor cells or whether these cells are host derived remains unclear at this moment. The RM-4 staining (pan-macrophage marker) shows that transplanted lungs have in total fewer monocytic cells. Interestingly, nearly 100% of these cells are anti-inflammatory. As we used an allo-isogenic model, a mild immunologic reaction is accepted, simulating a good cross-match. Interestingly, IL10, a potent chemoattractant for anti-inflammatory macrophages is downregulated in Deguelin-treated animals. However, a downregulation of the anti-inflammatory cytokine IL10 is not contradictory. First, IL10 is mainly produced by antigen presenting cells (APCs). Immunocytochemistry showed that the infiltrating immune cells in the HIF-1 inhibitor-treated group are mainly anti-inflammatory. Therefore few APCs are recruited to the lung, resulting in a downregulated IL10 expression. Secondly, recent works demonstrated that IL10 must already be upregulated before the event to act as anti-inflammatory agent. Should it be upregulated after the inflammatory event, it has a pro-inflammatory effect [58]. Thirdly, IL4 is known to downregulate IL10 expression [59]. As IL4 is upregulated in Deguelin treated rats, the downregulation of IL10 is plausible. On the other hand, serum analysis of IL6 and IFNγ revealed, that control animals have significantly lower levels of IL6 and significantly higher levels of IFNγ. It is known that IL6 is not only a simple pro-inflammatory cytokine. Together with IL4, IL6 induces M2 polarization of macrophages, which reflects our findings [60]. The rather high level of IL6 in the sham group is plausible, as the thoracotomy leads to a rather large wound, which is known to upregulate IL6. Higher IL6 levels in Deguelin treated animals thus directly correlate with the better outcome. The low IL6 levels in the controls might also reflect the effect of IFNγ or might be explained by a different IL6 kinetics in this group. Comparatively IFNγ, that is known to inhibit M2 polarization in vivo, is upregulated in control animals, which show low levels of M2 macrophages [61]. Thus, this cytokine profile is plausible and reflects the pathways involved in M2 differentiation in this animal model. We think that for the initiation of a M2 polarization, some extent of inflammation must precede this reaction. Once macrophages are recruited to the site of inflammation, IL4, MRC-1 and CCL2 initiate M2 polarization. In the case of the controls, the initial IL6 upregulation is missing and thus, M2 differentiation is missing. Together with this, IL10, CXCR4 and ICAM-1 upregulation is leading to an overshooting inflammatory response. However, the exact mechanisms underlying this complex reaction remain unclear. We think it is rather a consequence of the cells infiltrating the grafts and the following specific reactions initiated by target-oriented cytokines. Therefore Deguelin is not likely to directly stimulate IL6, CCL2 or MRC-1.

Taken together, our data clearly indicate that in the model of orthotopic lung transplantation, a systemic treatment with Deguelin prior to transplantation is beneficial and reduces early complications such as PGD. The suppression of hypoxia induced pathomechanisms leads to an attenuated VEGF-A activity and therefore significantly reduces reperfusion edema. Deguelin treatment also prevents from pro-inflammatory phenotypes by suppressing ICAM-1 and CXCR4 expression. The exact mechanisms, by which Deguelin influences the CCL2, MRC-1 and IL4 signals are yet unknown and remain subject of future studies.

## Materials and Methods

### Ethics Statement

The study was approved by the Animal Care and Use Committee of the state of Hesse (Regierungspräsidium Darmstadt), Darmstadt, Germany (V54-19c20/15-F91/56). Surgery and animal care was performed in accordance with the “Guide for the care and use of laboratory animals=" (National Institutes of Health, volume 25, no. 28, revised 1996), EU Directive 86/609 EEC and German Protection of Animals Act.

### Deguelin Preparation and Administration

**Figure 10:**
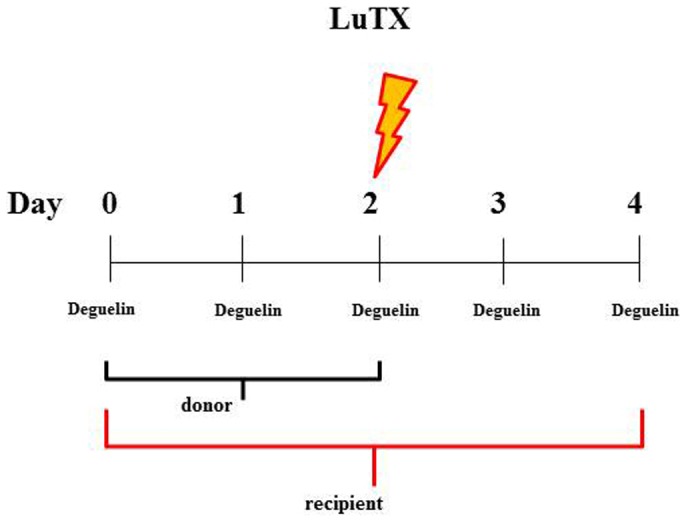
Therapeutic schedule. Scheme representing the therapy (gavages) and transplantation schedule. The lightning marks the day of transplantation. Experiment ends 48 hours after transplantation.

The HIF-1 inhibitor Deguelin was obtained from Enzo Life Sciences (Lörrach, Germany). Stock solution was prepared by dissolving Deguelin in 100% DMSO at 25 mg/ml and stored at −20°C until further use. For the use in animals, Deguelin was dissolved in corn oil at a final concentration of 10 mg/ml. Deguelin was applied via gavage twice a day at a dose of 4 mg/kg BW. Treatment started 3 days before transplantation in donors and recipients and ended 48 hours after transplantation (in recipients) (Fig. 10).

### Microvascular and Lung Epithelial Cell Hypoxia Assay

Human microvascular endothelial cells (HMECs) and human lung epithelial derived cells (HTB-177) (ATCC, Wesel, Germany) were cultured in 6 well plates (10e5 cells/well) to 80–90% confluence, using RPMI 1640 medium (PAA Laboratories, Pasching, Austria) plus 10% FCS. For the hypoxia assay, the medium was equilibrated at 37°C in 2% O2 and 5% CO2 for 24 h. 12 hours prior to onset of hypoxia, 100 nM final concentration of Deguelin was added to the cells. After 6 h of hypoxia (37°C, 2%O2, 5% CO2), cells were harvested and proteins were isolated. Experiments were performed in triplicate.

### Animals

Male Sprague Dawley rats (SD, Janvier, St. Berthevin, France) weighing 225–250 g were housed in the central research establishment of the Goethe-University Frankfurt. At the beginning of the experiment, rats were randomized to form pairs of donor and recipient rats, which were kept together in approved plastic cages (2 animals per cage), had water and food *ad libitum* and were housed in rooms equipped with a 12 h light cycle. The rats were individualized using a unique color code printed on the tail with water-resistant paint.

### Organ Transplantation and Harvest

In general the experiment was divided into two stages. The first stage comprised harvesting lungs from animals either treated or not treated with Deguelin under different ischemic conditions (Table 1).

**Table 1:** Layout of the first experimental stage.

Group Size	Treatment	Ischemic conditions
n = 6	Deguelin for 3 days	warm ischemia for 1 h
n = 6	untreated	warm ischemia for 1 h
n = 6	untreated	no ischemia (sham)

To harvest the lungs the rats were anesthetized with Ketamin-Xylazine (75/10 mg/kg body weight) and intubated. Anesthesia was continued with Isoflurane (1.5 vol %) using a pressure controlled small animal ventilator (Föhr Medical Instruments, Seeheim-Ober Beerbach, Germany) at 85 bpm and pmax = 25 mbar. Before removal, the lungs were flushed with 20 mL perfusion solution (Perfadex® solution, kept at 4°C) until the lungs turned pale. The left and right lungs were dissected from the heart-lung complex, one half of the obtained organs were snap-frozen in liquid nitrogen, the other half was stored in 4% formaldehyde. In the second stage of the experiments, an orthotopic allo-isogenic (SD-SD) unilateral lung transplantation was performed. These transplantation experiments compromised 2 groups: Deguelin-treated animals vs. control animals without treatment (Table 2, Fig. 10).

**Table 2:** Layout of the second experimental stage.

Group Size	Treatment	Animal
n = 6	Deguelin for 3 days preoperative	donor
	Deguelin for 3 days preoperative +2 days postoperative	recipient
n = 12	Untreated	controls (donor + recipient)

### Donor Procedure

Donor animals were anesthetized, intubated and ventilated as described earlier. Donor organs were obtained after perfusion of the heart-lung complex using an antegrade perfusion via the pulmonary artery. Lungs from the Deguelin group and control lungs were perfused each with 20 ml of Perfadex® (Vitrolife, Kungsbacka, Sweden), a solution with low potassium, not exceeding 20 cm H^2^O hydrostatic pressure. After removal, the heart-lung complex was wrapped in sterile gauze and kept on ice until backtable preparation.

### Backtable

For this purpose the left lung artery, vein and bronchus were dissected under sterile conditions from enclosing tissue. The loose ends of the three structures were drawn through cuffs (made from venous catheter), everted and fixed using a 6–0 prolene thread. The prepared left lung grafts were wrapped in sterile gauze and stored on ice until transplantation.

### Recipient Procedure

**Figure 11:**
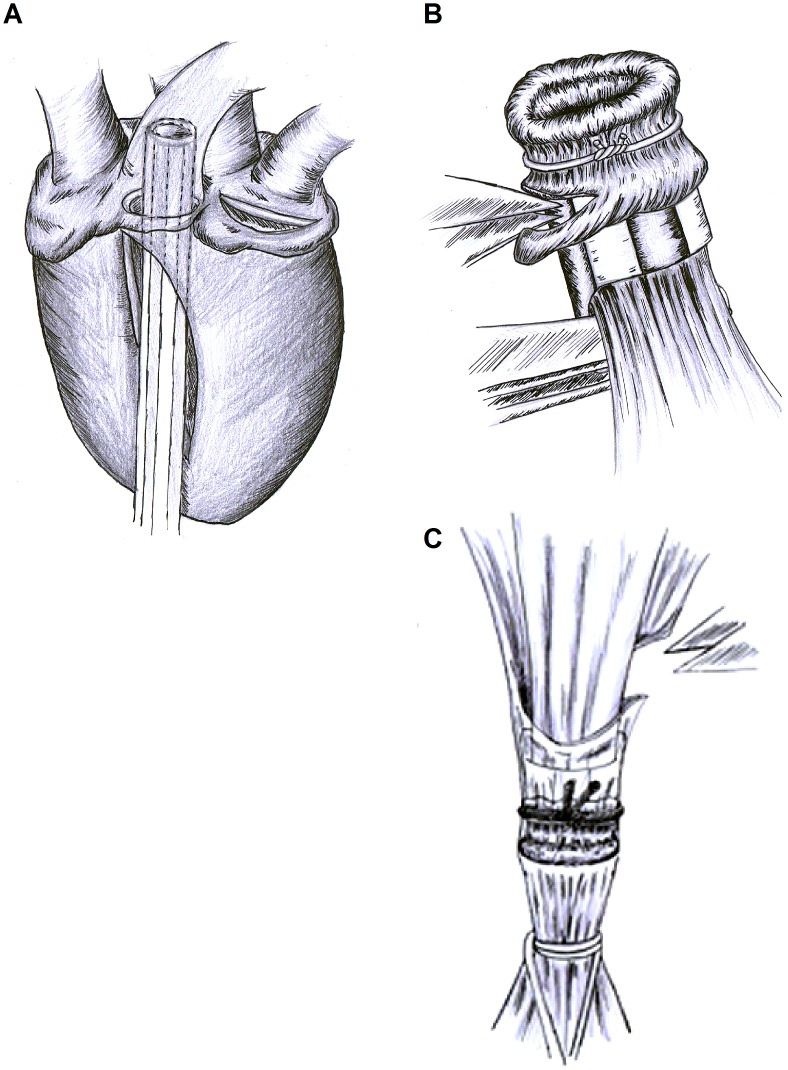
Technique of lung transplantation. (A) For perfusion, a venous catheter is placed through an incision in the right ventricle into the pulmonary artery. After perfusion, the heart-lung complex is removed *in toto*, suspended on the trachea that is ligated in maximal inspiration to avoid atelectasis. B) Cuffs are prepared using venous catheters. The vessel is slipped through the lumen and fixed with a 6–0 polypropylene suture. C) Donor (cuffed) blood vessels and bronchus were slipped (sleeved) into the recipient’s artery, bronchus and vein. When no twisting of the corresponding structure was observed, the anastomosis was secured with a 5–0 Silk suture placed around the cuff and sleeved recipient’s structure.

The recipient rats were anesthetized as described before. The thoracic cavern was opened using a left antero-lateral thoracotomy. The left lung lobe was carefully prepared, exposing the pulmonary artery, vein and bronchus. Then, the vessels and bronchus were selectively ligated using 6–0 prolene vessel loops to obstruct air and blood flow. Artery and vein were incised and flushed with a solution of Heparin/saline (150 U/mL). Then the bronchus was incised and the prepared donor lung was placed on top of the recipients left lung lobe. Connections between the structures of the donor organ and the recipient organism were established by inserting the corresponding donor structures into the incisions of the recipient vessels and bronchus. The generated connections were secured using 5–0 silk ligation (Fig. 11). The reperfusion of the vein and artery and the reestablishment of airflow through the bronchus was accomplished in the order mentioned. After successful transplantation the recipient’s left lung lobe was removed, the donor organ was placed into the thorax and the wound was closed. After successful suturing, local anesthesia was instilled (Ropivacain 0,2%, 1 ml) to ensure post-operative pain relief. Enrofloxacin was used as a post-surgical antibiotic and pain was controlled with Piritramid administered via drinking water (1,5 mg/50 ml). The recipient rats were observed for an additional 48 h and then killed under deep anesthesia. The Lungs were harvested, dissected and stored as described earlier.

### Wet-to-dry Ratio

A small part of the collected lungs was minced and stored in a reaction tube and the weight was determined. Then the tube was placed in an oven at 60°C for 72 h. Thereafter, the tube was re-weighed and the ratio between wet and dry weight was calculated. Comparison of the wet-to-dry ratios of different organ samples allowed drawing conclusions regarding the dimension of lung edema as reported elsewhere [22].

### RNA Isolation and PCR

RNA was isolated from homogenized lung samples using TriReagent solution (Sigma-Aldrich, USA) according to the manufacturer’s protocol. cDNA was synthesized using iScript cDNA Synthesis Kit (Bio-Rad Laboratories, USA). Realtime-PCR was performed using the StepOne Plus Realtime PCR (Applied Biosystems, USA) and following oligonucleotides: rCXCR4 forw. CACCAACAGCCAGAGCGCGA, rev. TGCGCTTCTGGTGGCCCTTG; rVEGF-A forw. CCAGGCTGCACCCACGACAG, rev.:CGCACACCGCCATTAGGGGCA; rICAM-1 forw. CGCAGTCCTCGGCTTCTGCC, rev. CGCAGTCCTCGGCTTCTGCC; rACTB forw. CTTGCAGCTCCTCCGTCGCC, rev. CTTGCTCTGGGCCTCGTCGC; rVCAM-1 forw. GGTGGCTGCACAGGTTGGGG, rev. ACCCACAGGGCTCAGCGTCA; rCD 163 forw. TGGGATCGCCGTGACGCTTC, rev. CAGCGACTGCCTCCACCGAC; rIL4 forw. GGCTTCCAGGGTGCTTCGCAA, rev. GTGGACTCATTCACGGTGCAGC; rCCL2 forw. GAGGCCAGCCCAGAAACCAGC, rev. GCAGCAGGTGAGTGGGGCATT.

### Protein Isolation, Western Blot and ELISA

Briefly, protein from left lungs was isolated as described earlier [23], [62]. 10% and 7.5% SDS-gels were loaded with 50 µg protein. Proteins were immunodetected on Hybond C supermembrane (Amersham Pharmacia Biotech, Buckinghamshire) with Spectra broad range marker (Fermentas, Germany) as a standard. The blots were probed with antibodies as follows: HIF-1 alpha - mouse monoclonal antibody (Abcam, Cambridge, UK), VEGF - mouse monoclonal antibody (Abcam, Cambridge, UK). Digitalization and evaluation of the blots was performed with a Kodak Imager (Carestream, Stuttgart, Germany). For serum sampling, blood was withdrawn and clotting was performed for 15 minutes followed by centrifugation with 3000 RPM for 10 minutes at 4°C. Serum supernatant was transferred to a new tube and then stored at −80°C for further processing. ELISA on rat IL6 and rat Interferon-γ (Thermo Fisher, Braunschweig, Germany) were performed according to the manufacturer’s protocol. Detection of the concentration was performed using a microplate reader (Bio-Tek Instruments, Bad Friedrichshall, Germany).

### Histology and Immunohistochemistry

One half of the samples from the left lung lobe was fixed in formaldehyde 4% for 48 h and dehydrated afterwards in solutions of increasing alcohol concentrations and then kept in methylbenzoate-I for 16 h and methylbenzoate-II for 8 h. After washing in liquid Paraffin (Merck, Darmstadt, Germany) samples were embedded in paraffin and cut to 4 µm slides. Samples were stained with haematoxylin and eosin (AppliChem, Darmstadt, Germany) according to the manufacturer’s protocol. Immunohistochemistry was performed with DAB-staining, using RM-4 pan-macrophage/dendritic cell marker (TransGenic Inc., Kobe, Japan), CD 163 (AbD Serotec, Düsseldorf, Germany), CD 68 (Millipore, Schwalbach, Germany), ICAM-1 (Labomics, Nivelles, Belgium) and CXCR4 (Abcam, Cambridge, UK) antibodies [62]. Briefly, after deparaffinization, the slides were rehydrated in decreasing alcohol concentrations. After blocking with 6% serum (from the species of the secondary antibody), the primary antibody was incubated for 2 h at room temperature. For detection, a secondary antibody labelled with HRP (anti mouse IgG, DAKO, Hamburg, Germany) (for CD 163, CD 68 and ICAM-1) was incubated for 1 h at room temperature. For detection of CXCR4 a biotinylated anti rabbit antibody was incubated for 1 h prior to detection with streptavidin-HRP (AXXORA, Lörrach, Germany). Development was performed with DAB incubation for 10 minutes and counterstaining was done with haematoxylin for 10 min. Images were taken with the Leica DM5000B microscope and analyzed using an automatized MatLab algorithm. The software determines the number of pixel in each RGB channel, thus measuring the relative area occupied by positive staining per image. Quantification was done setting brown pixels proportioned to total number of pixel.

### Statistical Analysis

Statistical analysis was performed with GraphPad Prism® 5.02 software (GraphPad Software, Inc., San Diego, California). Results are expressed as means ± standard error of the mean (SEM). Statistical significance was calculated using one-way ANOVA followed by Student’s t test. Statistical significance was set to p<0.05.
